# Editorial: Image-based planning of electric neurological treatments

**DOI:** 10.3389/fnhum.2022.1089818

**Published:** 2023-01-10

**Authors:** Reuben R. Shamir, Leo Joskowicz, Hagai Bergman

**Affiliations:** ^1^Novocure Ltd., Haifa, Israel; ^2^School of Computer Science and Engineering, The Hebrew University of Jerusalem, Jerusalem, Israel; ^3^The Edmond and Lily Safra Center for Brain Sciences, The Hebrew University of Jerusalem, Jerusalem, Israel; ^4^Department Medical Neurobiology, Institute for Medical Research, IMRIC, Hebrew University School of Medicine, Jerusalem, Israel

**Keywords:** tumor treating fields, deep brain stimulation, simulation, therapy planning, image based modeling

Tumor treatment fields (TTFields), deep brain stimulation (DBS) and other electric-based therapies have become the standard-of-care for the management of brain diseases including glioblastoma multiform (GBM), Parkinson's disease (PD), and epilepsy among others. While a comprehensive assessment of the actual biological effects of these therapies is still an ongoing research area, correlations to treatment outcomes have been reported, and a variety of software packages for image-based planning of electric neurological treatments have been implemented. This Research Topic incorporates four studies that report on recent advances in this field.

Cao et al. present new guidelines for burr hole surgery in combination with TTFields for glioblastomas. They incorporate an image-based simulation and optimize the TTFields dose for array layout planning. Their results suggest that burr hole surgery may result in a higher intensity of TTFields, especially when the tumor is in the vicinity of the skull.

Gentilal et al. report a study on the variations of temperature and impedance during TTFields treatment. Their study combines an image-based simulation with patients' log files that incorporate the actual temperature and impedance of the electrode arrays on the patients' skin. They provide practical suggestions for an improved placement of TTFields arrays.

Holtzman Gazit et al. present a novel post-operative GBM segmentation method with estimation of pixel-wise uncertainty for patient-specific modeling in simulation studies. The authors incorporate the uncertainty in a custom software that was evaluated by three experts. They conclude that the presented method performance is sufficient for the planning of TTFields in GBM patients.

Finally, Nordenström et al. have developed a Deep Brain Stimulation (DBS) planning system that optimizes the stimulation parameters to reduce rigidity as much as possible. To this end, they method incorporates retrospective monopolar reviews and image-based simulation. Their experimental results suggest that the presented method outperforms the classical method that targets the center of the subthalamic nucleus.

We congratulate the above authors for these achievements. We thank all the eight teams that have submitted a manuscript or an abstract for their efforts and participation and we hope to see their manuscripts, improve, revised and getting published soon.

In his book “My Inventions,” Nikola Tesla writes that “*Invention is the most important product of man's creative brain. The ultimate purpose is the complete mastery of mind over the material world, the harnessing of human nature to human needs.”* We hope that this Research Topic is a humble step in that direction and that you will find it valuable.

## Author contributions

RS has prepared the first draft. LJ, HB, and RS revised it together. All authors contributed to the article and approved the submitted version.

